# Effects of Health Qigong Program on Sleep Quality in Older Adults with Sleep Disturbance

**DOI:** 10.3390/healthcare14121661

**Published:** 2026-06-11

**Authors:** Jiayi Li, Fulong Shang, Moran Lyu, Qingyi Wang, Xiaohan Wang, Yuliu Tao

**Affiliations:** School of Physical Education and Sports, Soochow University, Suzhou 215021, China; 2306403025@stu.suda.edu.cn (J.L.); sd001231@163.com (F.S.); 20244006002@stu.suda.edu.cn (M.L.); 20244206028@stu.suda.edu.cn (Q.W.); xiaohanwang0903@126.com (X.W.)

**Keywords:** Health Qigong, sleep quality, older adults, self-reported sleep disturbance, actigraphy, controlled study

## Abstract

**Background/Objectives:** Sleep disturbance is common in older adults and is associated with impaired daily functioning and poorer health-related outcomes. This study examined whether a 12-week Health Qigong program could improve subjective and objective sleep quality in older adults with self-reported sleep disturbance. **Methods:** Sixty adults aged 55 years or older who reported poor sleep and had Pittsburgh Sleep Quality Index total scores above 5 were randomly allocated to an intervention group or a control group (30 participants per group; 10 men and 20 women in each group). Both groups received health education and lifestyle guidance, and the intervention group completed supervised Health Qigong sessions twice weekly. Sleep quality was assessed before and after the intervention using the Pittsburgh Sleep Quality Index and wrist actigraphy. **Results:** Baseline characteristics were comparable between groups. In adjusted post-intervention comparisons, the intervention group showed lower Pittsburgh Sleep Quality Index scores, higher sleep efficiency, less wake after sleep onset, fewer awakenings, shorter average awake time, and longer total sleep time than the control group. The adjusted group difference for sleep onset latency was not significant. **Conclusions:** These preliminary findings suggest that Health Qigong may be a feasible community-based practice for improving sleep quality in older adults with self-reported sleep disturbance. The overall pattern was more consistent with improvement in sleep continuity than in sleep onset latency, although this interpretation should remain cautious because no direct contrast between sleep maintenance and sleep initiation was tested.

## 1. Introduction

Sleep disturbance is common in later life and is associated with fatigue, mood problems, impaired daily function, and poorer quality of life [1]. In China, poor sleep quality is also highly prevalent in older populations [2]. Recent community-based evidence further indicates that poor sleep quality and short sleep duration are associated with poorer self-reported health among middle-aged and older adults [3]. Because long-term sleep medication use may increase dependence and adverse effects in older adults, practical nonpharmacological sleep health strategies remain relevant [4,5].

Exercise and mind-body interventions are increasingly supported as sleep quality interventions in older adults [6,7,8,9,10,11,12,13]. Recent evidence also supports exercise-based interventions as feasible nonpharmacological approaches for sleep improvement in populations with chronic health conditions [14]. Tai Chi and related traditional Chinese exercise programs appear especially promising because they combine gentle movement, breathing regulation, and attentional focus, and prior trials have reported sleep-related benefits in older adults [15,16,17,18,19,20,21]. Health Qigong may be particularly suitable for older adults because it is low impact, can be delivered in groups, and is feasible in community settings.

Recent randomized trials have further extended this evidence to more vulnerable older adults, showing improved sleep efficiency and quality of life after a 12 week Tai Chi program in older adults with mild to moderate cognitive impairment, and improved sleep quality and cognitive function when Tai Chi Chuan was augmented by 1 Hz rTMS in older adults with sleep disorders and mild cognitive impairment [22,23].

The role of Health Qigong in older adults with self-reported sleep disturbance remains insufficiently defined. Few studies have examined this population with both subjective sleep assessment and wrist actigraphy in the same controlled design [24,25,26]. In the present study, sleep continuity was used to describe the maintenance and stability of sleep after sleep onset. This concept included sleep efficiency, wake after sleep onset, awakenings, total sleep time, and average awake time. Sleep initiation was represented by sleep onset latency. The present study evaluated whether a 12-week Health Qigong program was associated with changes in subjective and objective sleep quality in older adults with self-reported sleep disturbance. It also examined whether the pattern of findings was more consistent with changes in sleep continuity than with changes in sleep initiation.

## 2. Materials and Methods

### 2.1. Study Design and Ethics

This study used a randomized parallel-group controlled intervention design with a 1:1 allocation ratio. Recruitment took place at a rehabilitation institution and in the community in Suzhou, China.

The study protocol was approved by the Ethics Committee of Soochow University under approval number SUDA20250425H02 on 25 April 2025. Written informed consent was obtained from all participants before study participation. The study was not preregistered in a public trial registry.

### 2.2. Participants and Eligibility

Participants were adults aged 55 years or older with self-reported sleep disturbance. Eligibility required subjective poor sleep and a Pittsburgh Sleep Quality Index (PSQI) total score >5 [27]. Participants were not diagnosed with insomnia disorder or another specific sleep disorder through a structured clinical assessment. A formal medical diagnosis of a sleep disorder was not required, and sleep apnea and restless legs syndrome were not specifically screened. Exclusion criteria included severe cardiopulmonary, musculoskeletal, neurological, or psychiatric disease; recent major life events; and other conditions that could interfere with safe exercise participation or sleep assessment.

### 2.3. Participant Flow and Sample Size

**Figure 1:**
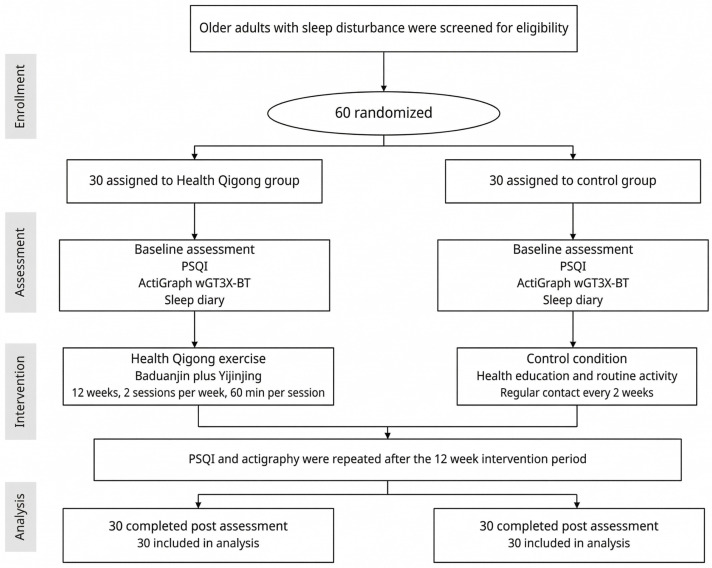
Study flow diagram. Baseline and post-intervention assessment included the PSQI, wrist actigraphy, and sleep diaries.

A total of 60 eligible participants entered group allocation. Participant flow, assessment, intervention, and analysis are shown in Figure 1. Complete baseline and post-intervention outcome data were available at both time points, and all participants were retained in the final analysis.

No a priori sample size calculation was available because this was an exploratory controlled study. A post hoc sensitivity analysis indicated that a total sample of 60 participants provided 80% power at α=0.05 to detect effects of approximately Cohen’s f=0.37 or larger, which corresponds roughly to partial $\eta^{2}=0.12$.

### 2.4. Group Allocation and Masking

Participants were individually randomized in a 1:1 ratio to the Health Qigong group or the control group. An independent researcher generated the allocation sequence using a random number table. The assignments were placed in sequentially numbered, opaque, sealed envelopes. After eligibility confirmation and baseline assessment, the next envelope in sequence was opened to assign the participant. This procedure maintained allocation concealment until group assignment.

Outcome data collection was not fully blinded. The staff involved in PSQI collection and ActiGraph fitting or retrieval were aware of the intervention context. However, raw ActiGraph files and sleep diaries were retained, and post-collection actigraphy processing was performed with anonymized participant codes by a researcher who did not have access to group allocation information. This procedure reduced bias risk for objective actigraphy outcomes, but the PSQI remained a participant-reported outcome and should not be considered blinded. Participants were not informed of the comparative study hypotheses or expected direction of between-group effects.

### 2.5. Intervention and Control Procedures

Both groups received health education and lifestyle guidance. Participants were recruited from a rehabilitation institution and fixed community settings, where daily routines were relatively stable. During the 12-week intervention, all participants were instructed to maintain their usual diet, sleep–wake schedule, medication use, and daily activities and to avoid additional structured exercise or new sleep-related interventions outside the study plan. Regular contact was maintained to monitor general routine changes, and no major changes in caffeine intake, daytime napping, medication use, additional physical activity, or apparent psychological status were reported or observed.

The control group did not receive a structured exercise program. Study staff contacted control participants every 2 weeks by telephone or in person follow-up to monitor sleep, activity, and general daily routine. This comparison condition provided basic health contact but was not designed as an active exercise or attention-matched control condition.

The intervention group completed a 12-week Health Qigong program based on Baduanjin and Yijinjing. The program used standardized Health Qigong routines issued by the General Administration of Sport of China, and a teaching team with unified professional training delivered the sessions according to a preset course schedule to maintain consistency in intervention content and teaching procedures. Sessions were delivered in small groups twice per week, and each session lasted 60 min. The instructor had more than 5 years of professional experience, more than 2 years of Health Qigong teaching experience, and national second-level qualifications as both a social sports instructor and referee. Before formal intervention, participants completed a 2-week familiarization period to learn the movement sequence, breathing coordination, and safe execution of the exercises. Each session comprised a 10 min warmup, 45 min main practice, and 5 min cooldown. The mean attendance was 22.6 of 24 supervised sessions, corresponding to a mean attendance rate of 94.0%. Attendance ranged from 20 to 24 sessions, and all participants attended at least 80% of the sessions.

Training emphasized coordinated movement, breathing regulation, and focused attention. Attendance was recorded during all supervised sessions. These logs were used to describe intervention adherence.

### 2.6. Outcome Measures

Outcomes were assessed at baseline and again after the 12-week intervention period.

Subjective sleep quality was assessed with the Pittsburgh Sleep Quality Index (PSQI) [27]. The PSQI contains 19 self-report items and yields a total score from 0 to 21, with higher scores indicating poorer sleep quality. Sleep diaries were also collected to support actigraphy scoring.

Objective sleep quality was assessed with the ActiGraph wGT3X-BT accelerometer (ActiGraph LLC, Pensacola, FL, USA). Wrist actigraphy is widely used for longitudinal sleep assessment in clinical and research settings [28,29]. Participants wore the device on the wrist continuously for 7 consecutive days at each assessment. Raw activity counts were collected in 60 s epochs, and bedtime and wake time recorded in daily sleep diaries were entered into ActiLife to define rest intervals for sleep scoring. The Cole–Kripke algorithm was used for actigraphy-based sleep estimation [30]. The device recorded wrist movement across the 24 h cycle, and these activity counts were converted into sleep–wake estimates and summary sleep parameters.

The actigraphy outcomes analyzed in the present study were sleep efficiency, wake after sleep onset, number of awakenings, sleep onset latency, total sleep time, and average awake time. Sleep efficiency, wake after sleep onset, number of awakenings, total sleep time, and average awake time were treated as indicators of sleep continuity, whereas sleep onset latency was treated as an indicator of sleep initiation.

### 2.7. Statistical Analysis

All statistical analyses were performed using SPSS version 26.0 (IBM Corporation, Armonk, NY, USA).

Continuous variables are reported as mean ± SD, and gender is reported as *n* (%). Normality and variance homogeneity were checked with the Shapiro–Wilk test and Levene’s test, respectively. Baseline group comparisons for age and sleep quality outcomes were examined with independent sample *t* tests to describe comparability at study entry. Gender was summarized descriptively.

The primary efficacy analysis was based on adjusted between-group comparisons of post-intervention outcomes. ANCOVA was used for each outcome. The post-intervention value was the dependent variable. Group was the fixed factor, and the corresponding baseline value was the covariate. This approach estimated the intervention effect while accounting for baseline outcome levels.

Before running each ANCOVA model, the homogeneity of the regression slopes was examined by testing the group-by-baseline interaction. Residual diagnostics were also examined. These diagnostics included residual distributions and standardized residuals for potential outliers. They were used to guide cautious interpretation of the adjusted results. Adjusted group differences, 95% CIs, *F* values, *p* values, and partial $\eta_{p}^{2}$ were reported.

Within-group changes were examined with paired-sample *t* tests. These analyses were descriptive and supportive only. They were not used as the primary evidence of intervention efficacy.

Additional effect estimates were reported to aid interpretation. Hedges’ *g* was calculated for baseline between-group differences. Cohen’s $d_{z}$ and 95% CI values were calculated for within-group changes. Cohen’s $d_{z}$ values of 0.2, 0.5, and 0.8 were interpreted as small, medium, and large. Partial $\eta_{p}^{2}$ values of 0.01, 0.06, and 0.14 were interpreted as small, medium, and large. To limit type I error across the 7 post-intervention group comparisons, Benjamini–Hochberg false discovery rate correction was applied. All tests were two-sided, and statistical significance was defined as *p* < 0.05.

Because all participants completed both assessments, complete case analysis was used and no imputation was required.

## 3. Results

### 3.1. Participant Status and Baseline Comparability

All 60 participants completed baseline and post-intervention assessments and were included in the analysis. Each group contained 30 participants. Table 1 shows baseline characteristics. Gender distribution was identical across groups, with 10 men and 20 women in each group. The mean age was 63.03 ± 4.22 years in the control group and 62.10 ± 3.91 years in the Health Qigong group.

No significant baseline differences were observed for PSQI or any actigraphy-derived sleep quality outcome (Table 2). Hedges’ *g* values ranged from −0.23 to 0.17, indicating trivial between-group differences at study entry.

### 3.2. Objective Sleep Outcomes

The primary adjusted between-group analyses favored the Health Qigong group for sleep efficiency, wake after sleep onset, number of awakenings, average awake time, and total sleep time. They did not show a significant group difference for sleep onset latency. Adjusted group differences were 3.25 percentage points for sleep efficiency (*p* < 0.001, $\eta_{p}^{2}$ = 0.177), −10.58 min for wake after sleep onset (*p* < 0.001, $\eta_{p}^{2}$ = 0.230), −1.70 awakenings (*p* < 0.001, $\eta_{p}^{2}$ = 0.189), −0.35 min for average awake time (*p* = 0.042, $\eta_{p}^{2}$ = 0.070), and 32.21 min for total sleep time (*p* < 0.001, $\eta_{p}^{2}$ = 0.554). The adjusted difference for sleep onset latency was −0.99 min (95% CI −3.33 to 1.35, *F* = 0.717, *p* = 0.401).

After false discovery rate correction across the 7 post-intervention group comparisons, the adjusted *p* values remained significant for sleep efficiency, wake after sleep onset, number of awakenings, total sleep time, average awake time, and PSQI, whereas sleep onset latency remained non-significant.

**Figure 2:**
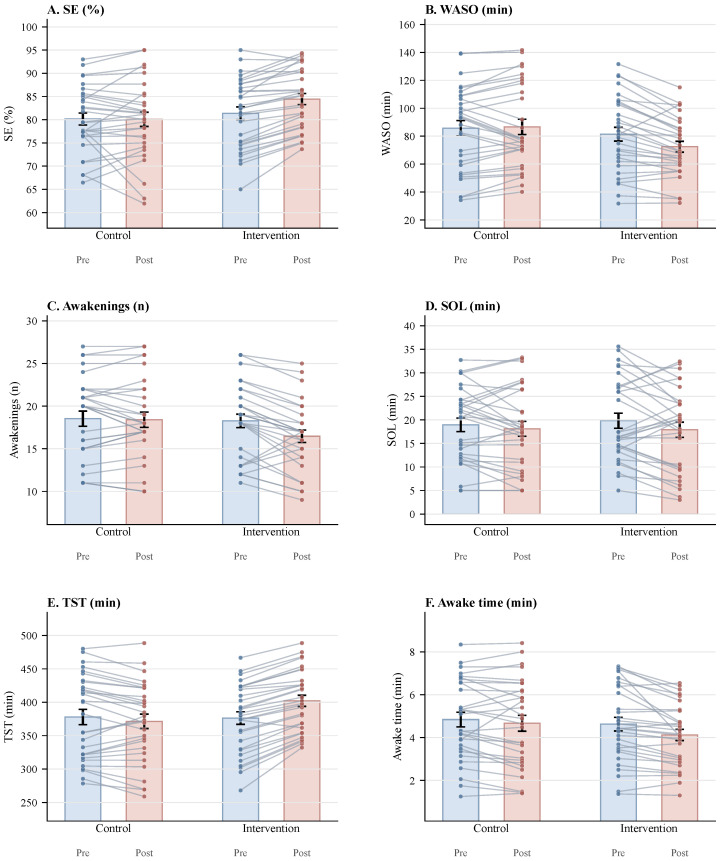
Objective sleep quality outcomes in the control group and the Health Qigong group before and after the intervention. Bars show mean ± SEM. Dots represent individual participants. Gray lines connect paired pre-test and post-test observations. Panels (**A**–**F**) show sleep efficiency, wake after sleep onset, number of awakenings, sleep onset latency, total sleep time, and average awake time.

Within-group analyses are reported only to describe the direction of change over time. Descriptively, the control group showed no significant change. In the Health Qigong group, sleep efficiency and total sleep time increased, whereas wake after sleep onset, number of awakenings, sleep onset latency, and average awake time decreased. Objective sleep quality outcomes are summarized in Table 3 and illustrated in Figure 2, where panels A to F show sleep efficiency, wake after sleep onset, number of awakenings, sleep onset latency, total sleep time, and average awake time.

### 3.3. Subjective Sleep Outcome

**Figure 3:**
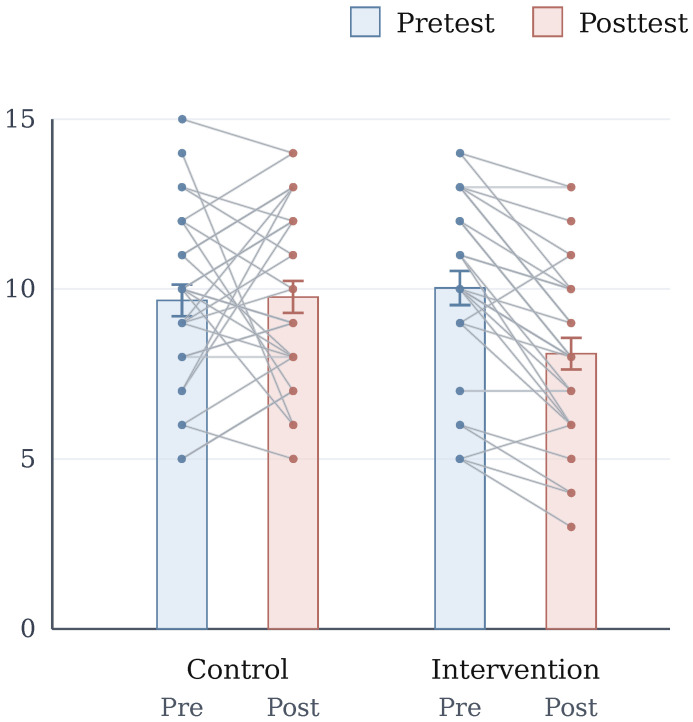
Subjective sleep quality outcome in the control group and the Health Qigong group before and after the intervention. Bars show mean ± SEM. Dots represent individual participants. Gray lines connect paired pre-test and post-test observations.

The primary adjusted between-group difference for PSQI was significant. The adjusted group difference was −1.87 points (95% CI −2.97 to −0.78, *F* = 11.746, *p* = 0.001, $\eta_{p}^{2}$ = 0.171), and the result remained significant after false discovery rate correction. Within-group analyses were descriptive only. PSQI remained stable in the control group but decreased in the Health Qigong group. The subjective sleep quality results are summarized in Table 4 and illustrated in Figure 3.

Taken together, the adjusted results favored the Health Qigong group for subjective sleep quality and for a subset of objective sleep quality outcomes, particularly sleep efficiency, wake after sleep onset, and total sleep time.

## 4. Discussion

The main finding of this study was that assignment to Health Qigong was associated with better subjective sleep quality and several objective sleep quality outcomes in older adults with self-reported sleep disturbance. In adjusted between-group comparisons, the Health Qigong group showed better outcomes for PSQI, sleep efficiency, wake after sleep onset, number of awakenings, average awake time, and total sleep time. Group differences were not significant for sleep onset latency. This interpretation was based mainly on adjusted between-group comparisons. Within-group pre–post comparisons cannot rule out temporal effects. They also cannot rule out regression to the mean or nonspecific study participation effects. They were therefore used only to describe the direction of change. The overall pattern was more consistent with partial improvement in sleep continuity than in sleep initiation. However, because no formal contrast directly compared sleep continuity outcomes with sleep initiation outcomes, this pattern should be interpreted as descriptive rather than definitive.

These findings are broadly consistent with recent evidence supporting exercise and mind–body practice as sleep quality interventions in older adults [10,11,12,13]. Recent meta-analyses have shown that Tai Chi and related exercise programs can improve PSQI scores in community-dwelling older adults and that exercise dose may influence the size of benefit [10,11]. Our results are also consistent with earlier controlled studies showing benefits for sleep quality or related health outcomes after Tai Chi, silver yoga, Baduanjin, and other exercise interventions [18,19,20,21,24,25,26,31]. The present study adds to this literature by evaluating subjective and objective sleep quality within the same controlled design and by focusing specifically on Health Qigong in older adults with self-reported sleep disturbance. This contribution should be viewed as incremental rather than definitive, because it extends existing mind-body exercise evidence to a symptomatic older sample while retaining the limitations of a modest, single-city controlled study.

The pattern across outcomes helps clarify the practical meaning of the results. Sleep efficiency, wake after sleep onset, number of awakenings, average awake time, and total sleep time improved in directions consistent with less fragmented and more sustained sleep. Sleep onset latency showed a modest within-group decrease in the intervention group but no significant adjusted group difference. This pattern is compatible with a stronger effect on some aspects of sleep continuity than on the speed of sleep onset, but it should not be overstated as proof of a selective mechanism.

The mechanism remains uncertain. Health Qigong may improve sleep through gentle movement, breathing regulation, attentional control, and behavioral self-regulation. These components may reduce presleep arousal and support more stable sleep across the night. This explanation is consistent with recent evidence on mind–body exercise, sleep regulation, and insomnia-related hyperarousal [10,11,12,13,32,33,34,35]. However, the present study did not directly measure autonomic, endocrine, neural, or psychological mediators central to behavioral sleep regulation. These mechanisms should therefore be regarded as possible explanations rather than confirmed mechanisms.

The PSQI score improved after the intervention. The adjusted between-group difference was 1.87 points. This finding should be interpreted with caution. A clear minimal clinically important difference for PSQI has not been established in older adults with self-reported sleep disturbance. Therefore, the PSQI change should be viewed as preliminary evidence of possible subjective sleep benefit. Some effect sizes were large. This was especially true for total sleep time. The sample size was modest. Large effects in small samples may be unstable or overestimated. Testing multiple sleep outcomes also increases interpretive complexity, even after false discovery rate correction. These results need confirmation in larger studies.

This study has several strengths. It combined subjective and objective sleep quality outcomes and evaluated a feasible group-based intervention for older adults. Several limitations should also be considered. The sample was modest and drawn from a single city, and no follow-up assessment was available, so the durability of the observed changes is unknown. Participants should be interpreted as older adults with self-reported sleep disturbance rather than as patients with clinically diagnosed insomnia disorder. Sleep disturbance was identified by subjective poor sleep and PSQI score rather than by a structured diagnostic assessment. The sample may therefore have included heterogeneous sleep complaints. These may have included difficulty initiating sleep, sleep maintenance problems, early awakening, poor perceived sleep quality, or other nonspecific sleep concerns. This heterogeneity limits interpretation and reduces generalizability to specific sleep disorder populations. Although the control group received health education, lifestyle guidance, and regular follow-up contact, the Health Qigong group received additional supervised group practice, instructor interaction, movement-based activity, and social engagement. Therefore, nonspecific effects such as attention, expectancy, social interaction, and increased daytime activity may have contributed to the observed improvements. Blinding was limited, as is common in behavioral exercise interventions. Outcome data collection was not fully blinded, and PSQI was self-reported. Blinded processing of coded ActiGraph data may reduce bias for objective sleep estimates, but it cannot remove expectancy or reporting bias in subjective outcomes. Attendance logs suggested high intervention adherence, but dose–response effects were not analyzed in the present report. Although the intervention followed standardized routines and a preset course schedule, no formal independent fidelity rating was conducted. Caffeine intake, daytime napping, medication use, additional physical activity, and psychological status were monitored only through routine contact and were not systematically assessed using standardized diaries or validated questionnaires; therefore, residual confounding cannot be fully excluded. Psychological mediators central to behavioral sleep regulation were not assessed with validated measures. These include anxiety, stress, depression, and related psychological variables. The study did not directly measure physiological mechanisms. Also, the sample size did not allow reliable subgroup analyses by sex or age. Future studies should include validated psychological measures, larger multicenter samples, longer follow-up, active control conditions, and public trial registration when feasible.

## 5. Conclusions

This study provides preliminary evidence that Health Qigong may improve subjective sleep quality and selected objective sleep outcomes in older adults with self-reported sleep disturbance. The findings should not be interpreted as direct clinical recommendations for insomnia disorder or other specific sleep disorders. Larger multicenter studies are needed. Future studies should include active control groups, blinded outcome assessment when feasible, longer follow-up, validated psychological measures, and direct measures of potential mechanisms.

## Figures and Tables

**Table 1:** Participant characteristics.

Variable	Control	Intervention	Diff	95% CI	Stat	*p*	ES
Age, years	63.03 ± 4.22	62.10 ± 3.91	−0.93	[−3.04, 1.17]	*t* = 0.889	0.378	*g* = −0.23
Gender: Male	10 (33.3)	10 (33.3)	—	—	—	—	—
Gender: Female	20 (66.7)	20 (66.7)	—	—	—	—	—

Values are mean ± SD or *n* (%). Diff = group difference. ES = effect size.

**Table 2:** Baseline sleep quality outcomes.

Outcome	Control	Intervention	Diff	95% CI	Stat	*p*	ES
SE (%)	80.15 ± 7.09	81.38 ± 7.56	1.23	[−2.56, 5.02]	*t* = −0.650	0.518	*g* = 0.17
WASO (min)	85.69 ± 30.12	81.42 ± 26.94	−4.27	[−19.04, 10.50]	*t* = 0.579	0.565	*g* = −0.15
Awakenings (n)	18.53 ± 4.88	18.27 ± 4.31	−0.27	[−2.65, 2.11]	*t* = 0.224	0.823	*g* = −0.06
SOL (min)	18.95 ± 7.90	19.84 ± 8.78	0.89	[−3.42, 5.20]	*t* = −0.413	0.681	*g* = 0.11
TST (min)	377.93 ± 62.65	376.33 ± 51.29	−1.60	[−31.19, 27.99]	*t* = 0.108	0.914	*g* = −0.03
Awake time (min)	4.84 ± 1.87	4.63 ± 1.74	−0.21	[−1.15, 0.72]	*t* = 0.458	0.649	*g* = −0.12
PSQI total	9.67 ± 2.54	10.03 ± 2.76	0.37	[−1.00, 1.74]	*t* = −0.536	0.594	*g* = 0.14

Values are mean ± SD. SE = sleep efficiency. WASO = wake after sleep onset. SOL = sleep onset latency. TST = total sleep time. Diff = group difference. ES = effect size.

**Table 3:** Objective sleep quality outcomes before and after the intervention.

Outcome	Control						Intervention						ANCOVA				
	Pre	Post	Chg	**95% CI**	p	$d_{z}$	Pre	Post	Chg	**95% CI**	p	$d_{z}$	Adj Diff	95% CI	F	p	$\eta_{p}^{2}$
SE (%)	80.15 ± 7.09	80.08 ± 8.41	−0.07	[−1.78, 1.64]	0.935	−0.02	81.38 ± 7.56	84.44 ± 6.51	3.06	[2.21, 3.92]	<0.001	1.33	3.25	[1.39, 5.11]	12.23	<0.001	0.177
WASO (min)	85.69 ± 30.12	86.65 ± 30.23	0.96	[−3.14, 5.05]	0.636	0.09	81.42 ± 26.94	72.46 ± 21.22	−8.97	[−12.90, −5.03]	<0.001	−0.85	−10.58	[−15.72, −5.44]	16.99	<0.001	0.230
Awakenings (n)	18.53 ± 4.88	18.40 ± 4.91	−0.13	[−0.74, 0.47]	0.654	−0.08	18.27 ± 4.31	16.47 ± 3.98	−1.80	[−2.57, −1.03]	<0.001	−0.87	−1.70	[−2.63, −0.76]	13.28	<0.001	0.189
SOL (min)	18.95 ± 7.90	18.10 ± 8.63	−0.85	[−2.33, 0.64]	0.252	−0.21	19.84 ± 8.78	17.91 ± 8.83	−1.93	[−3.83, −0.03]	0.047	−0.38	−0.99	[−3.33, 1.35]	0.72	0.401	0.012
TST (min)	377.93 ± 62.65	371.40 ± 59.04	−6.53	[−13.51, 0.46]	0.066	−0.35	376.33 ± 51.29	402.19 ± 45.65	25.86	[21.02, 30.70]	<0.001	2.00	32.21	[24.54, 39.87]	70.86	<0.001	0.554
Awake time (min)	4.84 ± 1.87	4.67 ± 2.03	−0.17	[−0.41, 0.07]	0.149	−0.27	4.63 ± 1.74	4.12 ± 1.43	−0.50	[−0.76, −0.24]	<0.001	−0.72	−0.35	[−0.69, −0.01]	4.31	0.042	0.070

Values are mean ± SD unless otherwise indicated. Intervention = Health Qigong. Chg = change. Adj diff = adjusted difference. $\eta_{p}^{2}$ = partial eta squared.

**Table 4:** Subjective sleep quality outcome before and after the intervention.

Outcome	Control						Intervention						ANCOVA				
	Pre	Post	Chg	95% CI	p	$d_{z}$	Pre	Post	Chg	95% CI	p	$d_{z}$	Adj Diff	95% CI	F	p	$\eta_{p}^{2}$
PSQI total	9.67 ± 2.54	9.77 ± 2.58	0.10	[−0.99,1.19]	0.853	0.03	10.03 ± 2.76	8.10 ± 2.55	−1.93	[−2.57,−1.30]	<0.001	−1.14	−1.87	[−2.97,−0.78]	11.75	0.001	0.171

Values are mean ± SD unless otherwise indicated. Intervention = Health Qigong. Chg = change. Adj diff = adjusted difference. $\eta_{p}^{2}$ = partial eta squared.

## Data Availability

The data presented in this study are not publicly available because they contain information that could compromise participant privacy. Deidentified data may be available from the corresponding author on reasonable request, subject to ethical and institutional approval where applicable.
